# Indocyanine-Green-Loaded Liposomes for Photodynamic and Photothermal Therapies: Inducing Apoptosis and Ferroptosis in Cancer Cells with Implications beyond Oral Cancer

**DOI:** 10.3390/pharmaceutics16020224

**Published:** 2024-02-04

**Authors:** Wei-Ting Liao, Dao-Ming Chang, Meng-Xian Lin, Jeng-Woei Lee, Yi-Chung Tung, Jong-Kai Hsiao

**Affiliations:** 1 Department of Medical Imaging, Taipei Tzu Chi General Hospital, Buddhist Tzu-Chi Medical Foundation, New Taipei City 23142, Taiwan; r05b42035@ntu.edu.tw (W.-T.L.); tch36363@tzuchi.com.tw (M.-X.L.); 2 School of Medicine, Tzu Chi University, Hualien 97004, Taiwan; 3 Research Center for Applied Sciences, Academia Sinica, Taipei 11529, Taiwan; dmchang@gate.sinica.edu.tw; 4 Department of Biomedical Sciences and Engineering, Tzu Chi University, Hualien 97004, Taiwan; jwlee@mail.tcu.edu.tw

**Keywords:** indocyanine green (ICG), liposome, photodynamic therapy, ferroptosis, apoptosis

## Abstract

Oral cancer represents a global health burden, necessitating novel therapeutic strategies. Photodynamic and photothermal therapies using indocyanine green (ICG) have shown promise due to their distinctive near-infrared (NIR) light absorption characteristics and FDA-approved safety profiles. This study develops ICG-loaded liposomes (Lipo-ICGs) to further explore their potential in oral cancer treatments. We synthesized and characterized the Lipo-ICGs, conducted in vitro cell culture experiments to assess cellular uptake and photodynamic/photothermal effects, and performed in vivo animal studies to evaluate their therapeutic efficacy. Quantitative cell apoptosis and gene expression variation were further characterized using flow cytometry and RNA sequencing, respectively. Lipo-ICGs demonstrated a uniform molecular weight distribution among particles. The in vitro studies showed a successful internalization of Lipo-ICGs into the cells and a significant photodynamic treatment effect. The in vivo studies confirmed the efficient delivery of Lipo-ICGs to tumor sites and successful tumor growth inhibition following photodynamic therapy. Moreover, light exposure induced a time-sensitive photothermal effect, facilitating the further release of ICG, and enhancing the treatment efficacy. RNA sequencing data showed significant changes in gene expression patterns upon Lipo-ICG treatment, suggesting the activation of apoptosis and ferroptosis pathways. The findings demonstrate the potential of Lipo-ICGs as a therapeutic tool for oral cancer management, potentially extending to other cancer types.

## 1. Introduction

Oral cancer is the 11th most common cancer worldwide, with higher rates in South Asia, Southeast Asia, and parts of Eastern Europe [1]. As a result of the co-influence of socioeconomic, genetic, and environmental factors, oral cancer exhibits a disproportionate impact on specific populations, potentially exacerbating social welfare concerns. The 5-year survival rate of oral cancer is only 50–66% [2]. Consequently, more treatment efforts should be studied to overcome health disparities. Multimodality treatment for oral cavity cancer is crucial because it involves a combination of therapeutic approaches to provide the most effective treatment while minimizing side effects and preserving functions. The primary treatment modalities for oral cavity cancer include surgery, radiation therapy, and chemotherapy, with some patients also benefiting from targeted therapy or immunotherapy [3]. Photodynamic therapy (PDT) is a minimally invasive treatment modality that has shown potential in the management of oral cancer because of its synergistic effect with other treatments. Moreover, PDT has shown promise in the treatment of oral pre-malignant lesions, which are often concurrently seen in oral cancer patients [4].

Among the various photosensitizers employed in PDT, ICG has garnered increasing interest in recent years. ICG, a tricarbocyanine dye, is a pharmaceutical compound that has had approval from the U.S. Food and Drug Administration (FDA) for over six decades. It has been broadly exploited for the assessment of hepatic function and used as a contrast agent for retinal vasculature imaging [5]. Compared with the predominantly utilized photosensitizer, 5-aminolevulinic acid (5-ALA), which exhibits excitation and emission within the visible light spectrum, ICG demonstrates excitation and emission within the near-infrared wavelength range [6]. This characteristic facilitates a significantly enhanced penetration depth in human tissues, consequently potentially leading to a more profound tumor eradication capacity [7,8]. Previous research conducted in our laboratory has revealed that ICG can induce ferroptosis in cancer cells when exposed to infrared light, and its photothermal effect after NIR irradiation has also been reported [9]. The multiple anti-cancer treatment possibilities offered by this single fluorescent dye have caught our attention, and we believe there may be undiscovered cancer treatment effects worth exploring.

Despite ICG’s multifaceted potential for tumor eradication, in vivo studies have demonstrated that its rapid uptake by plasma proteins hinders the accumulation of ICG within tumor tissues [10]. In contrast, liposomal ICG formulations provide unique benefits compared to molecular ICG structures stemming from their altered pharmacokinetic profiles, which include extended circulation durations and diminished clearance rates, attributable to their larger size and capacity to elude immune recognition [11]. Moreover, liposomal drugs can penetrate tissues and target cells that may be inaccessible to small-molecule drugs. One example of such a formulation is Doxil, which is a liposomal formulation of the chemotherapy drug doxorubicin. Due to its increased potency and longer half-life, Doxil typically requires lower dosages than doxorubicin and reduces the risk of cardiotoxicity associated with the latter [12]. The success of Doxil has inspired numerous studies on liposomes, and currently, there are several ongoing investigations into ICG-loaded liposomes. There are many advantages of ICG-loaded liposome as an anti-cancer treatment formulation. ICG-loaded liposomes (Lipo-ICGs) have proven their stability, biocompatibility, and tumor-targeting and imaging capabilities [13]. Nevertheless, the effectiveness of Lipo-ICGs has yet to be validated within the context of oral cancer. Moreover, most of the PDT in oral cancer research is limited to in vitro studies, which cannot reflect the complexity of the living environment [14]. More efforts should be focused on in vivo animal studies to facilitate the incorporation of the results into clinical trials. 

In this study, we employ the SAS-LN oral cancer cell line as a model system to assess the efficacy of Lipo-ICGs both in vitro and in vivo. The SAS-LN cell line demonstrates cervical lymph node metastasis in animal models, accurately reflecting the presentation of oral cancer in humans [15]. Consequently, we posit that our findings could facilitate a more reliable translation into clinical trials.

## 2. Materials and Methods

### 2.1. Liposomal ICG Formulation and Characterization

The thin-film hydration method was used to synthesize liposomes, and we mixed 1,2-dipalmitoyl-sn-glycero-3-phosphocholine (DPPC), cholesterol, and 1,2-distearoyl-sn-glycero-3-phosphoethanolamine-N-[amino (polyethylene glycol)-2000] (DSPE-PEG2000) (DPPC, cholesterol, and DSPE-PEG2000 were purchased form Avanti Polar Lipids Inc., Alabaster, AL, USA) in a mass ratio of 3:1:1 with chloroform in a 50 mL glass round-bottom flask. A lipid thin film can be obtained using a rotary evaporator at 45 °C. Then, 1 mL of 500 μg/mL of an ICG (obtained from Daiichi Sankyo Propharma Co., Ltd., Tokyo, Japan, Takatsuki Plant., Nagahama, Japan) solution was added to 10 mg of lipid thin film. The ICG–lipid solution was vortexed for 30 s to suspend the lipid thin film, and the ICG–lipid solution was sonicated at 50 °C for 20 min in a water bath. The ICG–lipid solution was transferred into a syringe extruder with polycarbonate membranes with a pore size of 200 nm (WHA111106, Whatman Nuclepore Track-Etched Membrane, Sigma-Aldrich, Inc., St. Louis, MO, USA). The solution was extruded 11 times, and the Lipo-ICGs with diameters smaller than 200 nm could be obtained after the process. The ICG–lipid solution was dialyzed for 24 h using tubing (132580, Spectrum Spectra/Por 6 Standard RC Pre-wetted Dialysis Tubing, 8MWCO, Thermo Fisher Scientific, Waltham, MA, USA), and the fabricated Lipo-ICGs were stored in the dark at 4 °C. The particle size and dispersity of the Lipo-ICGs were determined by dynamic light scattering (DLS), and the ICG concentration was measured in an 80% methanol solution of A_785_ with a BioTek Synergy2 258626 spectrophotometer. The lipid concentration of the Lipo-ICGs was analyzed with the Stewart assay and measured using a SPECTROstar^Nano^ spectrophotometer. The Stewart assay was performed by mixing 1.5 mL of chloroform, 0.5 mL of sample solution, and 2 mL of 0.1 M FeCl_3_·6H_2_O (ferric chloride hexahydrate, Sigma-Aldrich, Inc.)/0.4 M NH_4_SCN (ammonium rhodanide, Sigma-Aldrich, Inc.), and the mixed solution was vortexed for 20 s and centrifuged at 300× *g* for 3 min, and then the concentration of A_485_ was measured with the spectrophotometer in a cuvette. The established standard curves to estimate the ICG and lipid concentrations based on the absorbance are shown in Figure S1 in the supplementary material.

### 2.2. Determination of Photothermal Effect of Lipo-ICGs

We meticulously distributed 250 µL of the Lipo-ICG solution, containing ICG at a concentration of 250 µg/mL, into 14 individual 500 µL centrifuge tubes. Subsequently, we subjected the solutions to irradiation with 780 nm light for an array of time intervals, specifically: 0 s, 30 s, 60 s, 90 s, 120 s, 150 s, 180 s, 210 s, 240 s, 270 s, 300 s, 10 min, 15 min, and 20 min. The temperature of the centrifuge tube was photographed with a thermal camera (E52, Teledyne FLIR, Wilsonville, OR, USA).

Following the irradiation process, we introduced 250 µL of PBS to each tube, ensuring thorough mixing of the solutions. We then proceeded to centrifuge the samples within the concentrated centrifuge tubes for a duration of 1 h at a gravitational acceleration of 4000 g while consistently maintaining a temperature of 4 °C within the centrifuge tube. In the final step, we carefully extracted the lower liquid layer from each tube, assessed the absorbance spectra, and employed a regression-based approach to determine the ICG concentration.

### 2.3. Cellular Drug Uptake of SAS-LN

SAS-LN cells were seeded into an 8-well chamber slide (Nunc Lab-Tek, Thermo Fisher Scientific), 5 × 10^4^ cells/well, and 100 μg/mL of Lipo-ICGs or free ICG was added to the chamber slide and incubated for 4 h at 37 °C and 5% CO_2_. The concentration of the Lipo-ICGs used in the experiments was calculated based on the amount of ICG encapsulated in the liposomes. The SAS-LN cells were cultured in a growth medium consisting of DMEM, a high glucose concentration (Gibco 11965, Thermo Fisher Scientific), a GlutaMAX supplement (35050061, Thermo Fisher Scientific), and 10% Fetal Bovine Serum (FBS) (Gibco 10082, Thermo Fisher Scientific). After treating the drugs for 4 h, we replaced the ICG-containing medium with a normal growth medium and then performed fluorescent microscopy imaging (DMi8, Leica Microsystems, Wetzlar, Germany). The SAS-LN cell line, the lymph node metastases oral cancer cell line, was gifted by Jeng-Woei Lee’s lab, department of Biomedical Sciences and Engineering, Tzu-Chi University, Hualien, Taiwan. The cell line was derived from the oral cancer cell line SAS with a potentiation of its lymph node metastatic capability [15]. In order to observe the cellular uptake of the liposomes into the cells, a cell-permeable fluorescence dye, LysoTracker (L7528, Invitrogen LysoTracker Red DND-99, Thermo Fisher Scientific), at a concentration of 50 nM, was used to stain the organelles within the cells for 30 min at 37 °C, following the protocols suggested by the manufacturer.

In addition, in order to test the PDT effect in vitro, the SAS-LN cell line was cultured in a 25T flask and treated with the Lipo-ICGs. Subsequently, half of the flask was exposed to a 780 nm light source generated by a high-power light-emitting photodiode (LED) (M780LP1, Thorlabs, Inc., Newton, NJ, USA) with a power of 300 mW for 30 min before being replenished with a normal growth medium to allow for a 24 h recovery period.

### 2.4. Flow Cytometry Analysis of PDT with Lipo-ICGs

The SAS-LN cells were seeded into 96-well cell culture plates, 2.5 × 10^4^ cells/well, and incubated for 24 h at 37 °C and 5% CO_2_, and different concentrations of Lipo-ICGs were added into the well. When performing the fluorescence microscopic imaging of the cells treated with the Lipo-ICGs, we found that it is challenging to image the cells treated with the liposomes at a concentration lower than 50 μg/mL. In addition, we did test the liposome treatment at a concentration higher than 100 μg/mL. However, we found that the fluorescence signals within the cells remained at a similar level. Therefore, we performed the experiments with liposome concentrations of 50 and 100 μg/mL and used empty liposomes (i.e., 0 μg/mL) as the control. The Lipo-ICGs were exploited to treat the cells for 4 h, and the ICG-containing medium was replaced by the normal growth medium. The cells were then exposed to 780 nm light for 0, 20, and 40 min. After the cells were treated with Lipo-ICGs and light, we incubated the cells in the normal growth medium for 24 h to recover. In order to measure the number of apoptotic cells, an Annexin V/PI (propidium iodide) double-staining kit (obtained from Elabscience Biotechnology Inc., Tokyo, Japan) was used. The flow cytometry analysis was performed with a flow cytometer (BD FACSVerse Cell Analyzer, Becton, Dickinson and Company, Franklin Lakes, NJ, USA) with excitation/emission wavelengths set at 491/516 and 565/574 for Annexcin V and PI fluorescence signal detection, respectively.

### 2.5. RNA Sequencing

The SAS-LN cell line was maintained in a DMEM medium at 37 °C with 5% CO_2_ for 1 day and treated with liposomes for 4 h, and the cell pellet was collected after the liposome and light treatments. There were 4 groups: a control without any treatment, empty liposomes, Lipo-ICGs, and Lipo-ICGs with the 780 nm light treatment. For the light treatment experiments, the high-power light-emitting photodiode (LED) was used. The cells were seeded in a 12-well plate (3515 Costar, Corning, Corning, NY, USA) with a density and a volume of 1 × 10^5^ (cells/well) and 1 mL, respectively. For the ICG–liposome-treated cells, liposomes with a concentration of 50 μg/mL were used. During the light treatment, the LED was placed on top of the well plate for 20 min to ensure the proper quality of the RNA collected after the treatment for the RNA sequencing. Total RNA was extracted and purified from the SAS-LN cell pellet using TRIzol™ Reagent and magnetic oligo-dT beads, and an RNA sequencing analysis was performed on an Illumina NovaSeq6000 platform. The data were analyzed by CASAVA base calling and stored in FASTQ format. The RNA sequencing analysis was completed by BIOTOOLS Co., Ltd., New Taipei City, Taiwan.

### 2.6. Tumor Cell Line-Derived Xenograft (CDX) Models

We obtained female BALB/cAnN.Cg-Foxnlnu/CrlNarl nude mice (aged 6–8 weeks) from the National Laboratory Animal Center (Taipei City, Taiwan). They underwent a subcutaneous injection of 1 × 10^6^ SAS-LN cells in 100 μL of DPBS into the bilateral flank. The tumor xenografts grew for 10 days, and we started the PDT by intravenously injecting Lipo-ICGs into the tail vein. The size of the xenografts was then measured daily. The animal study was approved by the Institutional Animal Care and Use Committee at Taipei Tzu Chi General Hospital (109-IACUC-014).

### 2.7. In Vivo Animal Imaging and PDT with Lipo-ICGs

The nude mice (4 for each experimental condition) were bearing tumor cells for 10 days and were intravenously injected with 15 mg/kg of Lipo-ICGs. After injecting Lipo-ICGs for 4 h, the nude mice were anesthetized with isoflurane and exposed to 780 nm near-infrared light for 15 min every day. The tumor volume was measured every day for 2 weeks, and the nude mice were humanely sacrificed. The tumor volume formula is as follows: tumor volume = 1/2[(tumor length) × (tumor width)^2^]. The differences in body weights and tumor volumes between the untreated and Lipo-ICG-treated mice were statistically analyzed based on a Student’s t-test.

## 3. Results

### 3.1. Synthesis of Lipo-ICGs

**Figure 1:**
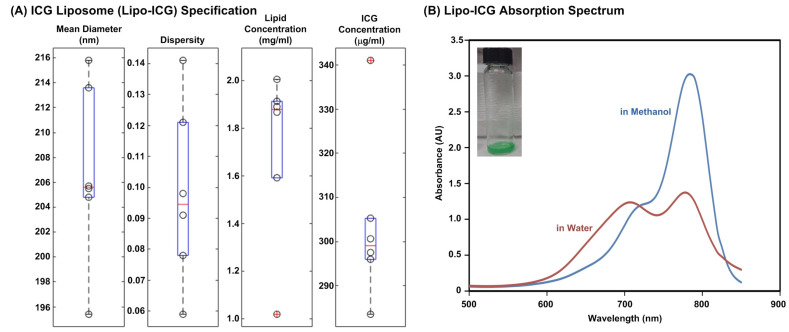
Lipo-ICG specification and absorption spectrum: (**A**) Lipo-ICGs synthesized by membrane filtration methods have a particle size of around 206 nm, which coincides with the pore size of the polycarbonate membranes used in the formulation process, 200 nm. The dispersity of Lipo-ICGs was about 0.095, meaning the molecular weights of the particles were very uniform. The lipid concentration was about 1.9 mg/mL, and the ICG concentration was about 300 μg/mL. (**B**) A quenching effect can be observed when Lipo-ICGs are dissolved in water.

We conducted a thorough characterization of the synthesized Lipo-ICGs to evaluate their quality (Figure 1). The Lipo-ICGs produced via membrane filtration techniques exhibited a particle size of approximately 206 nm, which aligns with the pore size of the 200 nm polycarbonate membranes employed during the formulation process. The dispersity index of the Lipo-ICGs was found to be approximately 0.095, indicating a highly uniform molecular weight distribution among the particles. The lipid concentration was determined to be approximately 1.9 mg/mL, while the ICG concentration was approximately 300 μg/mL. Notably, upon dissolving Lipo-ICGs in water, a quenching effect was observed. In addition, the ICG encapsulation efficiency was characterized as 81.74% by dividing the measured total ICG amount encapsulated within the liposomes by the total ICG amount used for the liposome fabrication. The amount of encapsulated ICG was measured by dissolving the Lipo-ICGs in 80% methanol for the concentration estimated according to the aforementioned light absorbance method. The total amount can then be calculated by multiplying the concentration by the solution volume.

### 3.2. Photothermal Effect of Lipo-ICG and ICG Release

**Figure 2:**
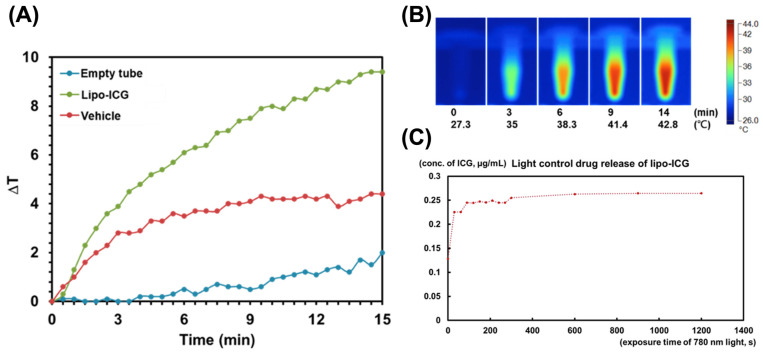
Observation of photothermal effect and triggered drug release. We documented a time-dependent photothermal effect: (**A**) the use of 780 nm light facilitated thermal effects on Lipo-ICGs. For comparison, a vehicle and an empty tube were utilized. A notable rise in temperature was seen within the Lipo-ICG group. (**B**) Temperature imaging demonstrated a heat map where the centrifuge tube’s temperature escalated to 42.8 degrees Celsius after being irradiated for a period of 14 min. This temperature increase subsequently triggered the release of the Lipo-ICG drug. (**C**) Shortly after the application of the 780 nm irradiation, the molecular form of ICG became detectable.

We also observed a time-sensitive photothermal effect in the experiments. The thermal response of the Lipo-ICGs was enhanced by their exposure to 780 nm light. Following 14 min of light irradiation, the temperature within the centrifuge tube increased to 42.8 °C, in comparison to a lesser rise to 29.3 °C in a test tube containing only PBS and to 33.8 °C in the test tube containing the vehicle (Figure 2A). Sequential heatmap images illustrating the progressive thermal impact on Lipo-ICGs due to light exposure are presented in Figure 2B. This thermal augmentation facilitated the further release of the Lipo-ICG drug. Not long after the initiation of the 780 nm irradiation, the molecular structure of ICG became detectable (Figure 2C).

### 3.3. Cellular Uptake of ICG and Lipo-ICGs

**Figure 3:**
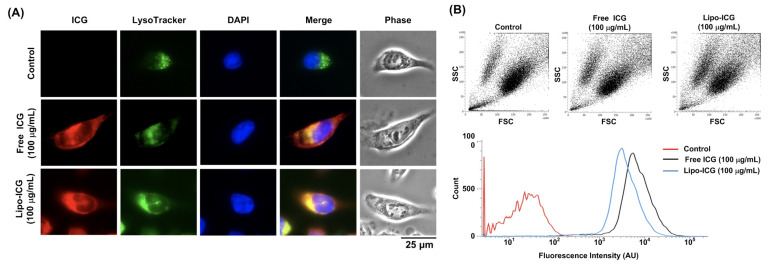
Fluorescent microscopy imaging and flow cytometry analyzing the cellular uptake of Lipo-ICGs or free ICG in the tongue squamous cell carcinoma line SAS-LN: The SAS-LN cell line was incubated in a growth medium with 100 μg/mL of Lipo-ICGs or free ICG at 37 °C and 5% CO_2_ for 4 h, which was then replaced with PBS to measure fluorescent signals through fluorescent microscopy imaging and flow cytometry. (**A**) Through fluorescent microscopy imaging, we observed that ICG signals could be traced in both the ICG and Lipo-ICG groups. Moreover, the intracellular ICG signal was co-located with LysoTracker, indicating that ICG can accumulate in lysosomes. (**B**) In ICG signal detection by flow cytometry using the APC-Cy7 channel, both Lipo-ICG and free ICG signals were detected, meaning the SAS-LN cell line successfully and efficiently ingested both Lipo-ICGs and the molecular form of ICG. There was no significant difference in uptake efficiency between the Lipo-ICGs and free ICG groups in the 2D culture.

Through fluorescent microscopy imaging (Figure 3), we observed ICG signals in both the ICG and Lipo-ICG treatment groups. To further investigate the subcellular localization of ICG accumulation, we found that the intracellular ICG signal co-localized with LysoTracker, suggesting that ICG accumulates within lysosomes. The internalization of both ICG and the Lipo-ICGs was further validated using flow cytometry, utilizing the fluorescent APC-Cy7 channel. ICG signals were detected in both the oral cancer cells treated with Lipo-ICGs and free ICG, indicating that the SAS-LN cell line efficiently internalized both Lipo-ICGs and the molecular form of ICG. The statistical analysis shows no significant difference in the uptake efficiency between the Lipo-ICG and free ICG groups in a conventional mono-layer cell culture.

### 3.4. In Vitro PDT Treatment Effect of Lipo-ICGs

**Figure 4:**
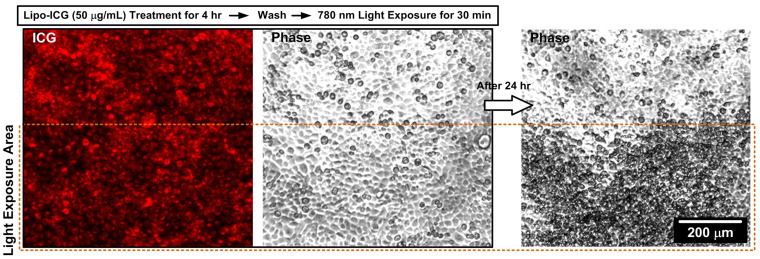
Fluorescent microscopy imaging of the Lipo-ICG PDT’s effects on the cells. The SAS-LN cell line was treated with Lipo-ICGs and kept in the 25T flask, and half of the flask was exposed to 780 nm light for 30 min and then replaced with the normal growth medium to recover for 24 h. Under fluorescent microscopy, there were fewer fluorescent signals in the light treatment area. Under light microscopy, a cell apoptosis phenomenon was observed in the light treatment area, where cells became rounded in shape or disintegrated into small pieces.

Under fluorescent microscopy, a reduction in fluorescent signals was observed within the light-treated area (Figure 4). Moreover, bright-field microscopy revealed evidence of cell apoptosis in the light-treated region, as indicated by cells adopting a rounded morphology or disintegrating into smaller fragments.

### 3.5. Flow Cytometric Analysis of Cell Apoptosis Using Annexin V/PI Staining

**Figure 5:**
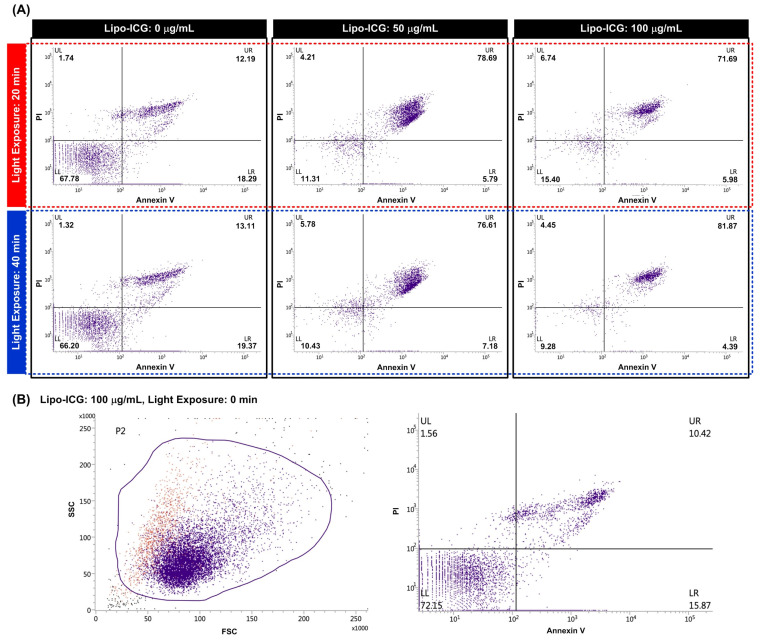
Flow cytometry analysis of photodynamic therapy with Lipo-ICGs. The SAS-LN cell line was incubated in a growth medium with 0, 50, and 100 μg/mL of Lipo-ICGs at 37 °C and 5% CO_2_ for 4 h, which was then replaced with the normal growth medium, exposed to 780 nm light for 0, 20, and 40 min, and then the normal growth medium was left to recover for 24 h. Annexin V/PI fluorescent signals of cell apoptosis analysis were seen by flow cytometry. A cell apoptosis phenomenon was shown in the light treatment groups. Moreover, there was a dose-responsive tendency in both light exposure time and Lipo-ICG dosage (**A**). In this experiment, the SAS-LN cells uptake Lipo-ICGs, and apoptosis appears after exposure to 780 nm light. The effectiveness of phototherapy was further verified by labeling SAS-LN cells without light treatment, as illustrated in (**B**); there are predominantly viable cells, not apoptotic cells, in the flow cytometry study.

Our study demonstrated light-induced toxicity for the cells treated using the liposomes containing ICG (Figure 5). We observed similar cell viability (66.2% and 67.8%) between the treated cells exposed to light for 20 and 40 min, respectively. The experiment results show that the cells treated with empty liposomes without ICG can live well under light exposure for up to 40 min. In contrast, for the cells treated with the liposomes containing ICG, we saw an increase in the dead cell population after the light exposure. We observed similar cell viability between the 50 μg/mL ICG–liposome-treated cells exposed to light for 20 and 40 min. For the cells treated with liposomes containing 100 μg/mL of ICG, the dead cell population increased from 71.69% to 81.87% when the light exposure time increased from 20 to 40 min. The results show an ICG concentration-dependent response for the cells exposed to light for 40 min and a light exposure time-dependent response for the cells treated with a relatively high ICG concentration (100 μg/mL). In addition, in order to confirm the cell compatibility of the ICG liposomes, we treated the cells with the liposomes containing 100 μg/mL of ICG without light exposure. The results (Figure 5B) show that the majority of the cells (>72%) are live, indicating the cell compatibility of the fabricated ICG liposomes.

### 3.6. Heatmap of RNA Sequence Data

**Figure 6:**
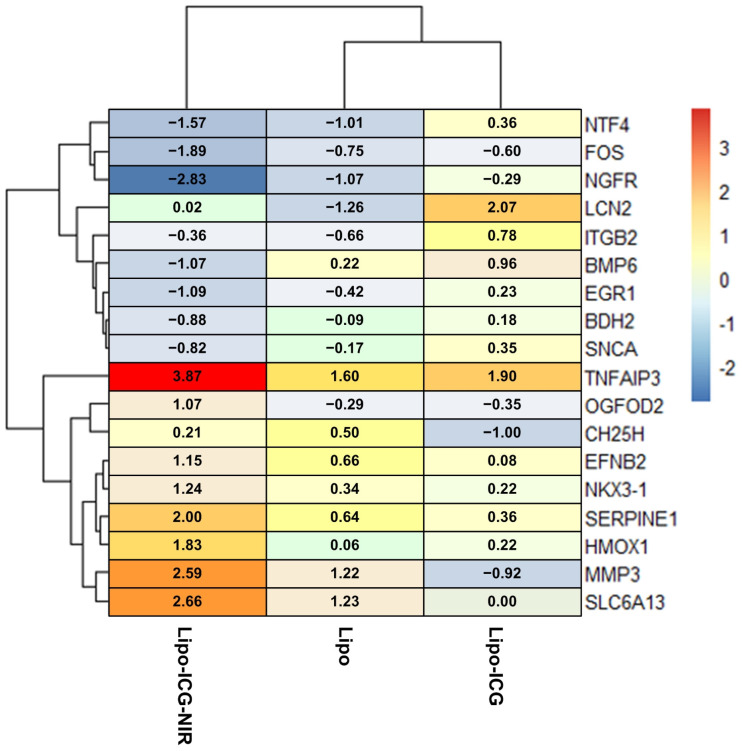
RNA-seq heatmap comparing oral cancer cells in the SAS-LN cell line subjected to three types of treatments: 1. uncoated liposomes (Lipo), 2. liposomes loaded with ICG (Lipo-ICG), and 3. liposomes loaded with ICG and combined with NIR treatment (Lipo-ICG-NIR). The lines display the results of a hierarchical cluster analysis, showing the similarity among the genes and samples. The results revealed two clusters in the heatmap, including up-regulated and down-regulated clusters. When comparing the Lipo-ICG-NIR group with the Lipo and Lipo-ICG groups, the RNA sequences *TNFAIP3*, *OGFOD2*, *CH25H*, *EFNB2*, *NKX3-1*, *SERPINE1*, *HMOX-1*, *MMP3*, and *SLC6A13* in the Lipo-ICG-NIR group showed overexpression, while *NGFR*, *FOS*, *NTF4*, *LCN2*, *ITGB2*, *BMP6*, *EGR1*, *BDH2*, and *SNCA* showed underexpression.

The heatmap data delineated two primary clusters, corresponding to genes with overexpression and underexpression. Upon comparing the Lipo-ICG-NIR group with both the Lipo and Lipo-ICG groups, distinct patterns of gene expression were observed. The RNA sequences for *TNFAIP3*, *OGFOD2*, *CH25H*, *EFNB2*, *NKX3-1*, *SERPINE1*, *HMOX-1*, *MMP3*, and *SLC6A13* exhibited overexpression in the Lipo-ICG-NIR group. Conversely, the genes *NGFR*, *FOS*, *NTF4*, *LCN2*, *ITGB2*, *BMP6*, *EGR1*, *BDH2*, and *SNCA* were characterized by underexpression in the same group (Figure 6).

### 3.7. In Vivo Tumor Suppression Effect following Near-Infrared-Treated Lipo-ICG Therapy

**Figure 7:**
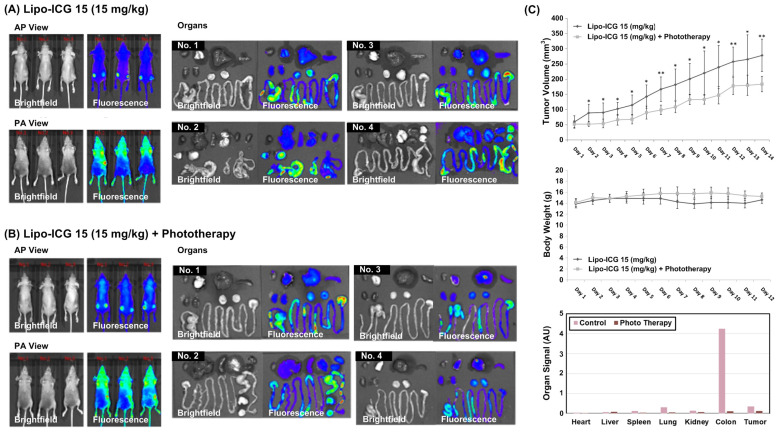
In vivo tumor growth suppression after photodynamic therapy. Oral cancer xenografts were established by bilateral flank injection of SAS-LN cells, and we used IVIS images for in vivo and ex vivo imaging. (**A**,**B**) The tumor xenografts could be imaged before the 780 nm laser irradiation, indicating the efficiency of Lipo-ICG delivery. The ex vivo IVIS imaging shows most of the ICG signals are detected in the colon, but there are still considerable fluorescent signals in the tumor xenograft. We measured tumor volume curves as illustrated. (AP: Posterior Anterior; PA: Anterior Posterior). (**C**) There was a slower growth rate in the 780 nm light treatment group than in the no irradiation group. The body weight curves showed no significant difference between the light treatment and no light treatment groups. Data are presented as mean ± standard deviation (n = 4). (*: *p* < 0.05; **: *p* < 0.01).

An oral cancer xenograft model was established through a bilateral flank injection of SAS-LN cells, and we employed in vivo and ex vivo IVIS imaging to visualize the results (Figure 7A,B). The successful visualization of the tumor xenograft prior to 780 nm laser irradiation highlights the efficiency of Lipo-ICG delivery. The ex vivo IVIS imaging revealed that the majority of ICG signals were detected in the colon; however, a substantial presence of fluorescence was still observed within the tumor xenograft. We further evaluated the tumor volume trajectories, as depicted in Figure 7C. The 780 nm light treatment group exhibited a reduced growth rate in comparison to the non-irradiated control group. Meanwhile, the body weight curves demonstrated no significant disparities between the light treatment and non-treatment groups. 

## 4. Discussion

We successfully synthesized liposomal ICG utilizing membrane filtration techniques. The in vitro assessment demonstrated both the effective labeling efficiency and cancer cell elimination capabilities of the Lipo-ICGs, suggesting their great potential for theranostic applications. Upon the exposure of the Lipo-ICGs to NIR irradiation, both the apoptotic pathway and cell necrosis were initiated. Furthermore, the thermal effects associated with Lipo-ICGs were observed to facilitate their deterioration and the release of free ICG, a crucial process after the liposomes are internalized within the cytoplasm. The RNA sequencing data revealed that Lipo-ICG PDT exhibits multifaceted therapeutic effects against oral cancer cells, including the promotion of apoptosis, the inhibition of metastasis, and the suppression of both cancer cell differentiation and proliferation. Most notably, our study demonstrated the targeting efficacy and treatment response of Lipo-ICGs in an animal xenograft model.

Through comprehensive RNA sequencing, we discerned the multi-faceted impact of Lipo-ICGs under near-infrared (NIR) treatment. The RNA sequencing heatmap illuminated the extensive range of effects when Lipo-ICGs are utilized in conjunction with NIR treatment. Significantly, the up-regulated TNFAIP3/A20 gene is potentially capable of instigating apoptotic proteins such as caspase-8 and caspase-3, fostering apoptosis. This finding aligns with previous research [16], implying a parallel role for the photosensitizer ICG. In addition, the positive association of TNFAIP3 with the promotion of ferroptosis [17] implies that Lipo-ICG phototherapy might employ both apoptosis and ferroptosis pathways. This was further reinforced by our prior study, which demonstrated that ICG in tandem with NIR encourages cancer cell ferroptosis through the down-regulation of GPX4 and SLC7A11 [9]. This extensive view of ICG light therapy in relation to ferroptosis could potentially offer a novel strategy for cancer treatment. We noted the up-regulation of the tumor suppressor gene NKX3-1 and the down-regulation of oncogenes FOS and NTF4, along with the down-regulation of the p53 inactivator NGFR, all indicating an inhibitory influence of ICG phototherapy on cancer cell proliferation and differentiation. Further substantiating this anti-proliferative proposition, we observed the up-regulation of the CH25H (cholesterol 25-hydroxylase) gene and the down-regulation of the proliferation promoter ITGB2. In addition, EGR1 and LCN2, which are known to instigate metastasis and angiogenesis, exhibited down-regulation, insinuating that Lipo-ICG phototherapy could counteract cancer metastasis. Hence, ICG phototherapy appears to mitigate cancer cell proliferation, metastasis, and angiogenesis while encouraging apoptosis and potentially ferroptosis, thereby impacting cancer progression.

At present, a myriad of potential applications for liposomes encapsulating ICG are undergoing development. One approach incorporates magnetic iron oxide nanoparticles within Lipo-ICGs, thus establishing a dual-imaging medium capable of fluorescence and magnetic resonance imaging [18]. However, validation of the treatment response remains to be accomplished. Another investigation employed liposomes encapsulating a high concentration of ICG to generate J-aggregates, which are used in photoacoustic imaging for the in vivo detection of ovarian cancer [19]. Yet, the therapeutic efficacy of this modality requires further validation. Concurrently, Lipo-ICGs have been engineered into a nano-based hydrogel integrated with doxorubicin, showing promise in cancer cell eradication in an in vitro murine breast cancer 4T1 model [20]. However, the absence of in vivo data necessitates additional investigation. The therapeutic potential of Lipo-ICGs has been reinforced via their integration with 1,2-dioleoyl-3-trimethylammonium-propane (DOTAP), with a photothermal effect validated both in vitro and in vivo [21]. However, the ongoing clinical trials of DOTAP pose limitations on its immediate availability for medical translation. The drug-release efficacy of Lipo-ICGs has been corroborated using calcein encapsulated within the Lipo-ICGs. Within a mere 15 s, calcein release was observed. Correspondingly [22], our study yielded analogous results, identifying free ICG within the same timeframe following light irradiation. These findings suggest that light-triggered drug release occurs with both small molecules, as evidenced in our model, and larger molecules, as demonstrated in the external study. Through our rigorous investigation conducted via an in vivo model, we have effectively demonstrated that the use of clinically available drugs can result in photodynamic, photothermal, and light-triggered drug release in addition to theranostic effects, thus underscoring their enhanced translatability.

In addition to the multifaceted anticancer role of Lipo-ICGs that is worth investigating, our study holds significant translational potential based on the in vivo animal experimental results. In contrast to many in vitro studies utilizing 2D cultures where cancer cells form a single layer, in vivo investigations more accurately represent clinical scenarios where the laser must penetrate multiple tissue layers to eradicate tumor cells. It is well established that capillaries, abundant in the tumor bed, can absorb light, consequently reducing the singlet oxygen (^1^O_2_) yield and diminishing PDT efficiency. Therefore, in terms of tissue penetration depth, the most effective photosensitizers are those with excitation and emission wavelengths within the near-infrared range [23]. In contrast to many clinically available photosensitizers, such as 5-aminolevulinic acid (5-ALA) or porfimer sodium, which utilize light in the red visible spectrum, ICG boasts superior excitation and emission wavelengths of 780 nm and 820 nm, respectively [24,25]. The longer wavelengths provide better penetration into tissues, which positions ICG as an ideal photosensitizer for cancer tissue treatment [26]. Our in vivo tumor imaging findings effectively showcase the benefits of ICG in enhancing the visualization of tumor structures. These results have potential implications for clinical practice, particularly in the context of tumor ablation procedures. By utilizing ICG-assisted visualization, clinicians can better target oral cancer tissues for laser irradiation while sparing normal tissues, thus minimizing potential damage to surrounding healthy tissue.

Our research also bears significant potential for rapid translation into clinical trials, particularly due to the integration of the PDT light source into the endoscopic system [27]. This integration facilitates access to hollow tubular structures, including, but not limited to, the oral cavity, esophagus, trachea, and bronchus, as well as the stomach and colon. It is noteworthy to mention that PDT has already been clinically employed for managing esophageal strictures arising from advanced esophageal cancer and airway obstructions induced by advanced lung cancer [28,29]. There is also growing evidence showing the efficacy of PDT in gastric cancer, colon cancer, or even biliary cancer clinically [30]. The use of Lipo-ICGs may present an intriguing avenue of research for mitigating the risk of skin burns, a notable adverse event associated with photosensitizer usage. Additionally, owing to their specific excitation and emission wavelength characteristics, Lipo-ICGs might also enhance therapeutic efficacy in the management of deep-seated cancerous tissues. With the development of fiber optics, PDT has also been used in thoracoscopes to treat pleural malignancies and laparoscopes to treat peritoneal metastasis [27,31]. Anticipated future investigations will likely encompass the exploration of Lipo-ICGs for their theranostic potential in the treatment of diverse cancer types.

The burgeoning interest in the advancement of ICG-incorporated liposomes has led to the identification of enhanced potential anticancer effects. A notable example is sonodynamic therapy (SDT), a therapeutic modality that synergistically utilizes ultrasound and a sonosensitizer to produce reactive oxygen species (ROS) for the targeted treatment of cancer [32]. In our prior research, we discovered that ICG alone can induce ferroptosis in cancer cells [9]. Our present study also indicates that Lipo-ICGs may exhibit a ferroptotic effect, as supported by the RNA-SEQ data. Investigating whether Lipo-ICGs demonstrate a ferroptotic effect during sonographic stimulation would be an intriguing line of inquiry. In addition, the Food and Drug Administration (FDA) has approved certain cancer-detecting pharmaceuticals derived from ICG, such as Pafolacianine [33]. This compound, a fusion of a folate analog and ICG, exhibits promising targeting capabilities for cancers rich in folate receptors. Given our study’s demonstration of the theranostic potential of liposomal ICG, it is anticipated that future research will focus on the investigation of Pafolacianine encapsulated within liposomes to further assess its theranostic effects.

The metabolism of our ICG-loaded liposomes is considered safe for humans. Our ex vivo study reveals that the majority of fluorescent signals are found in the gastrointestinal tract, suggesting that biliary secretion plays a significant role in ICG metabolism. Earlier research on similar liposomes containing PEGylated ICG, which were administered intravenously, showed visualization in the liver 24 h post-injection [34]. Our study provides further insight into the metabolic pathway of these ICG compounds, which we believe will ultimately be excreted through fecal matter. 

Although we successfully synthesized Lipo-ICGs, verified their chemical and physical characteristics, and tested them for their in vitro and in vivo anticancer effects, there are some limitations to our study. First, our study lacks a thorough assessment of the toxicity and potential side effects of Lipo-ICGs, specifically in relation to dose concentration. However, the primary treatment core of the Lipo-ICGs is ICG, which has been a medication for decades whose safety has been verified. Its potential toxicity is low. Second, the RNA sequencing data are limited, as RNA sequencing only provides a snapshot of gene expression at the time of sampling. However, the conclusions drawn about the effect of Lipo-ICGs on specific genes might not represent the full picture of Lipo-ICGs’s long-term impact, and the in vivo environment is also quite different from that of the in vitro study. In addition, our subcutaneous xenograft model does not represent the clinical situation in which oral cancer is located, where the vasculature and mucosa coverage of the tumor may affect the PDT and drug delivery. There is also uncertainty in the light exposure parameters; the optimal light exposure parameters (wavelength, intensity, duration, etc.) for the clinical application of Lipo-ICGs were not established in our current study. Future research should be directed towards establishing the optimal conditions for Lipo-ICG treatments. This will involve the use of an orthotopic rat model that not only provides a reasonable light penetration depth but also exhibits vasculature characteristics akin to those of the human oral cavity. Such an approach will significantly enhance the translational relevance of our findings. Lastly, and most importantly, there is a lack of comparison with existing treatments; our study does not compare the efficacy of Lipo-ICGs with existing cancer treatment modalities, making it difficult to determine their potential advantage or superiority. Further thorough comparisons should be investigated.

## 5. Conclusions

Our study highlights the successful synthesis and characterization of liposomal ICG, displaying significant potential for oral cancer treatment applications. Through in vitro and in vivo analyses, we demonstrated its effective cellular uptake, apoptosis induction, and tumor growth inhibition, particularly in oral cancer. Lipo-ICGs’s PDT capabilities, paired with their safe metabolism, suggest a promising avenue for further exploration in various other cancer types, particularly those accessible via endoscopic systems. With the potential for broader theranostic use, our work lays the foundation for future clinical investigations.

## Data Availability

Data are available on demand.

## Supplementary Materials

The following supporting information can be downloaded at https://www.mdpi.com/article/10.3390/pharmaceutics16020224/s1: Figure S1: Standard curves for the measurements of ICG and lipid concentrations based on the optical absorbance.
