# Supplementary material for: Indocyanine-Green-Loaded Liposomes for Photodynamic and Photothermal Therapies: Inducing Apoptosis and Ferroptosis in Cancer Cells with Implications beyond Oral Cancer
Source: Pharmaceutics. 2024 Feb 4;16(2):224. doi: 10.3390/pharmaceutics16020224 (PMC10891763; doi:10.3390/pharmaceutics16020224)
Supplement: Supplementary file 1 [file pharmaceutics-16-00224-s001.zip › pharmaceutics-2784874-supplementary.pdf]

Supplementary Material

Indocyanine-Green-Loaded Liposomes for Photodynamic and Photothermal Therapies: Inducing Apoptosis and Ferroptosis in Cancer Cells with Implications beyond Oral Cancer

Wei-Ting Liao [1,2,†], Dao-Ming Chang [3,†], Meng-Xian Lin [1], Jeng-Woei Lee [4], Yi-Chung Tung [3,*] and Jong-Kai Hsiao [1,2,*]

1 Department of Medical Imaging, Taipei Tzu Chi General Hospital, Buddhist Tzu-Chi Medical Foundation, New Taipei City 23142, Taiwan; r05b42035@ntu.edu.tw (W.-T.L.); tch36363@tzuchi.com.tw (M.-X.L.)
2 School of Medicine, Tzu Chi University, Hualien 97004, Taiwan
3 Research Center for Applied Sciences, Academia Sinica, Taipei 11529, Taiwan; dmchang@gate.sinica.edu.tw
4 Department of Biomedical Sciences and Engineering, Tzu Chi University, Hualien 97004, Taiwan; jwlee@mail.tcu.edu.tw
* Correspondence: tungy@gate.sinica.edu.tw (Y.-C.T.); jongkai@tzuchi.com.tw (J.-K.H.);
Tel.: +886-2-2787-3138 or +886-2-2787-3162 (Y.-C.T.); +886-2-6628-9779 (ext. 61114) (J.-K.H.)
† These authors contributed equally to this work.

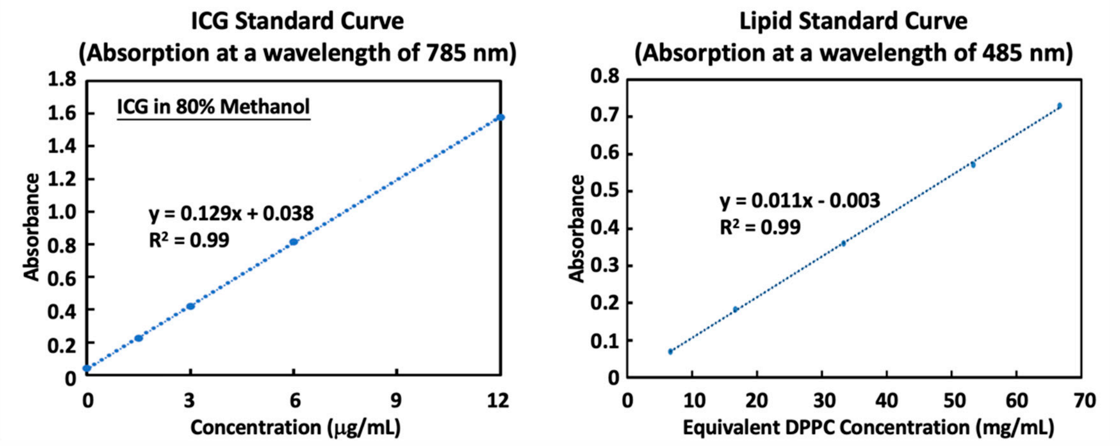

Figure S1. Standard curves for the measurements of ICG and lipid concentrations based on the optical absorbance.
